# Trends in Pediatric Neck Injuries During Sporting Activities

**DOI:** 10.3390/jcm13247713

**Published:** 2024-12-18

**Authors:** Victor M. Lu, Subaraman Ramchandran, Thomas Errico, Stephen George

**Affiliations:** 1 Department of Neurological Surgery, University of Miami, Miami, FL 33136, USA; 2 Department of Orthopedic Surgery, Center for Spinal Disorders, Nicklaus Children’s Hospital, Miami, FL 33155, USA

**Keywords:** pediatric, sport, neck, cervical, injury, emergency room

## Abstract

**Background:** Although neck injuries secondary to sporting activities in the pediatric demographic presenting to the emergency department (ED) are common, predictors of needing higher-level care (HLC) outside immediate treatment and release are not clear. The aim of this study was to describe how these neck injuries present in the United States (US) and identify predictors of HLC. **Methods:** We interrogated the US National Electronic Injury Surveillance System (NEISS) database for presentations to the ED of pediatric patients (aged 6–18 years old) whose primary complaint was neck injury in the setting of sport between 2014 and 2023. Statistical analysis utilized weighted estimates to evaluate incidence and then univariate and multivariate regression analyses were carried out to identify parameters associated with HLC for these patients. **Results:** Overall, a national weighted total of 360,885 ED presentations were estimated to have occurred in the last decade without any statistical change over the years. The mean age was 12.7 years, with more males (60.0%) than females (40.0%). The most common race observed was Black (50.6%), and these injuries most commonly occurred at a sporting complex (62.9%). Neck strains (59.9%) were the most common diagnosis seen across all presentations, followed by neck pain (29.1%), neck contusions (including abrasion) (6.4%), neck fractures (1.7%) and neck lacerations (0.8%). Ultimately, the majority of presentations were treated and released from the ED (95.5%). There were 4.5% of presentations, however, that did require HLC. Older age (OR 1.07, *p* = 0.004), male gender (OR 1.51, *p* = 0.002), involvement of other body part(s) (OR 1.45, *p* = 0.007) and non-strain neck injuries (OR 11.8, *p* < 0.001) were all independent, statistically significant predictors of HLC. Football (18.6%) was the most common sport associated with these presentations overall, but this was driven mostly by male cases. For females, the most common sport associated with these presentations was cheerleading (12.3%). **Conclusions:** In the last decade, neck injuries secondary to sporting activities in the pediatric demographic presenting to the ED have remained consistent. We have identified a unique set of predictors for presentations requiring HLC. These findings can be used in tandem with the findings that there are a number of gender-specific sports that drive these presentations to develop more sensitive and specific protocols for both primary prevention and ED triaging.

## 1. Introduction

Sports and sporting activities are pivotal components in child development—better mental health, decreased cardiovascular risks, reduction in overweight and obesity and higher bone density have all been associated with sports during childhood [1,2,3]. However, as with all activities that involve impact, there is an inherent risk of injury. Injury secondary to participating in sporting activities can vary greatly in terms of incidence and severity, and a better understanding of how these injuries occur and present are important for maintaining a healthy level of participation in sports for all pediatric patients [4].

Neck injuries, or injuries to the cervical spine, are not well understood in the setting of pediatric sports. Injuries during sports can sometimes be innocuous, resulting in paresthesia and pain on movement, or may have outcomes as serious as immediate paralysis that has the potential to be permanent in nature [5]. Mechanisms can include cervical fractures anywhere from cervical 1 to cervical 7, bony retropulsion leading to spinal cord injury and even spinal cord injury without radiographic abnormality (SCIWORA) [6]. These extreme situations mandate a higher level of care once such an injury presents, involving multiple specialties including orthopedics, neurosurgery, radiology and critical care.

The occurrence of these neck injuries in pediatric patients who participate in sports is poorly understood, partly due to the breadth of sports in which these injuries can occur. The aim of this study was to interrogate a national database of emergency department (ED) neck injury presentations secondary to sporting activities in pediatric patients with documented sports and disposition outcomes to define how these injuries present, identify predictors of disposition and identify the sports most commonly associated with these presentations. All these data will better assist future efforts in further reducing the risk of serious neck injuries in sports for all pediatric participants.

## 2. Methods

### 2.1. Database

We interrogated the National Electronic Injury Surveillance System (NEISS) database, as previously reported [7]. Briefly, the NEISS is managed by the Consumer Product Safety Commission (CPSC) [8]. This commission selects a sample of 100 hospitals across the US, which includes both academic and community hospitals. Each ED presentation in these hospitals is recorded, documenting patient demographics, injury diagnosis and details, location of injury, disposition and narratives. For the purpose of this study, all entries in the NEISS between 2014 and 2023 were included.

### 2.2. Selection Criteria

Cases were included if the patient (1) was of pediatric age to participate in organized sports, which, per the American Academy of Pediatrics (AAP), is recommended at ages 6 years and above, making our age range 6–18 years [9]; (2) had documented evidence of involvement in a sporting activity; and (3) had a documented mechanism of injury and final disposition from ED. Sporting activities within the NEISS database included archery, baseball/softball, basketball, biking/cycling, billiards or pool, bowling, boxing, curling, darts, fencing, fishing, football, golf, hockey, horseback riding activity, lacrosse, rugby, martial arts, mountain climbing, racquet sports, shuffleboard, skateboards, scooters, hoverboards, skating, soccer, swimming activity, track and field activities, trampolines, unicycles, volleyball, water skiing, tubing and surfing.

### 2.3. Data and Outcomes

As data were extrapolated from survey data, counts were rounded to the nearest integer value; as such, all sums were not expected to be equal. Demographic data included patient age, which was grouped into three categories (child (6–10 years), preadolescent (11–14 years) and adolescent (15–18 years)); gender; race; location of sport; diagnosis; injury type; disposition and sporting activity. Our primary outcome of interest was disposition status, which included treated and released, observation, transfer, admission and death. Dispositions outside treated and released were then categorized as higher-level care (HLC).

### 2.4. Statistics

Estimates of total counts and proportions, with their respective lower and upper 95% confidence interval (95% CI) bounds, were calculated using linear survey data analyses, utilizing the weighted estimate by the NEISS query database. Linear regression was used to estimate possible dichotomous trends over the last ten years. Regression analyses were used to identify statistically relevant predictors of HLC, with univariable analysis first conducted to identify candidate associations; based on these results, variables demonstrating a between-groups test statistic of *p* < 0.10 were included in multivariable analysis to determine the independence of these factors. All *p*-values were two-sided with significance defined as *p* < 0.05. All statistical analyses were conducted with STATA 16.1 (StataCorp, College Station, TX, USA).

## 3. Results

### 3.1. Overall Trends

**Figure 1:**
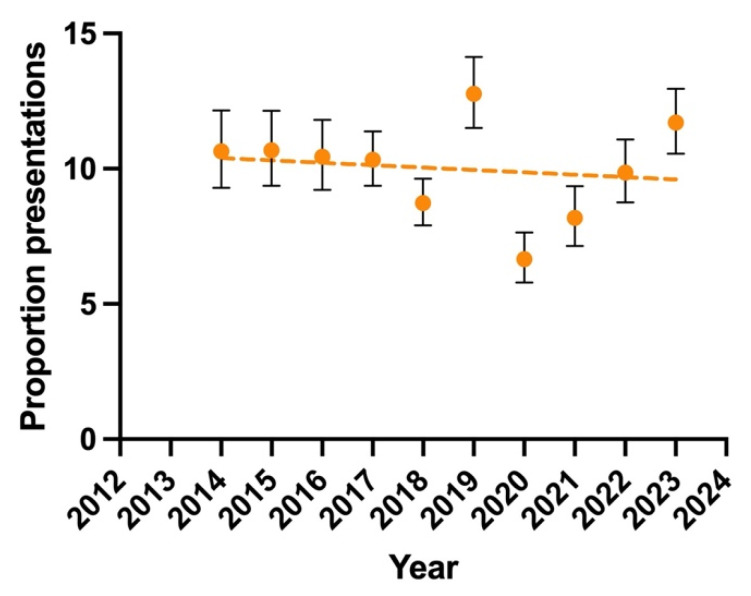
National weighted estimates for total number of ED presentations for pediatric neck injuries acquired during sporting activities in the United States by year. Data are presented as total (dot) and 95% CI (error bar) for each year, and linear regression (dotted line) for change over time (*p* = 0.675).

Overall, a national weighted total of 360,885 (95% CI, 276,200–445,600) presentations to the ED were estimated to have occurred in the United States in the last decade of 2014–2023 for pediatric neck injuries during sporting activities. By year, the most presentations occurred in 2019 (12.8%, 95% CI 11.5–14.3), and the least presentations occurred in 2020 (6.7%, 95% CI 5.8–7.6) (Figure 1). Linear regression did not detect a statistically significant change in the total number of cases presenting each year over the last decade (*p* = 0.675).

### 3.2. Demographics

For the entire cohort, the mean age was 12.7 years (95% CI 12.6–13.0), spread slightly more in common in both the preadolescent (36.3%) and adolescent (36.7%) age groups than in the child (27.0%) age group (Table 1). These presentations were more common in males (60.0%) than females (40.0%), and seen in more patients of Black (50.6%) race than White (32.2%) or Other (17.2%) races. Of all the injuries, they most commonly occurred at a sporting complex (62.9%), followed by at school (24.1%) and then at home (13.1%).

### 3.3. Injury Characteristics

Evaluation in the ED ultimately revealed that neck strains (including sprains) (59.9%) were the most common diagnosis seen across all presentations (Table 1). This was then followed by neck pain (29.1%), and more minor diagnoses included neck contusions (including abrasion) (6.4%), neck fractures (1.7%) and then neck lacerations (0.8%). Most of these injuries were in isolation to the neck only (75.9%), with a minority presenting with injury to other body part(s) (24.1%).

### 3.4. Disposition

Ultimately, the majority of presentations were treated and released from the ED (95.5%). There were 4.5% of presentations, however, that did require higher-level care (HLC)—the most common was admission to hospital (1.9%), followed by transfer to another care facility (1.6%), then observation (0.9%) and mortality (0.2%). Examples of mortality causes sampled included tracheal rupture and spinal cord injury.

### 3.5. Predictors of Higher-Level Care

Multivariable regression analysis demonstrated that older age (OR 1.07, 95% CI 1.02–1.13, *p* = 0.004), male gender (OR 1.51, 95% CI 1.17–1.95, *p* = 0.002), neck injury as well as other body part(s) (OR 1.45, 95% CI 1.11–1.88, *p* = 0.007) and non-sprain injuries (OR 11.8, 95% CI 7.65–18.2, *p* < 0.001) were all independent, statistically significant predictors of HLC being needed for these presentations in the ED. These were the only tested variables that demonstrated significance upon univariable testing. Dichotomous evaluation of the top 10 sports did not demonstrate any of them to be an independent predictor of HLC.

### 3.6. Sport Rankings

Overall, the most common sport associated with neck injuries presenting to the ED in the United States was football (18.6%) (Table 2). However, there was a clear gender bias, as this constituted the most common of presentations for all male cases (30.0%), and was outside the top 10 sports associated with these presentations in female cases (<1.0%). For females, the most common sport associated with these presentations was cheerleading (12.3%). Four (40%) of the top ten sports overall seen in the top ten sports across both genders (i.e., trampoline, basketball, soccer and cycling) featured in this analysis. Of note, trampoline was the second most common sport seen overall (9.9%), in both the male (9.8%) and female (10.1%) cohorts. Sports seen in the male top 10 not seen in the female top 10 included football, wrestling, baseball, weightlifting, ice hockey and lacrosse. Additionally, sports seen in the female top 10 not seen in the male top 10 included cheerleading, horse riding, softball, dancing and volleyball.

## 4. Discussion

In this study, we report the trends in presentations to the ED of pediatric patients presenting with concerns regarding neck injuries acquired during sporting activities. We demonstrate that the most common demographic was Black males in their preadolescence experiencing an injury during football while at a sporting complex. There are, however, many niches to consider within this wide age group, differences in gender and the breadth of sporting activities these patients can participate in.

Understanding how these presentations are managed is important from multiple perspectives. As a patient, appropriate identification of treatments versus unnecessary hospitalization is ideal. For surgical specialists, it allows them to better understand the nature of consultations that will present for their evaluation. As a hospital system, the need to utilize HLC is ideally minimized to those that truly require this care in order to improve system operations. Finally, as these are pediatric patients, there is a need to best triage these presentations in order to care for the parents and family that these patients present with.

In the US during the evaluated time period, there was no significant change in the annual average number of ED presentations across the country. The most common demographic features we found paralleled those of other sports injury presentations seen in pediatric patients—both male gender and Black race are also the most common in the settings of neurological concussion, [7] knee injury [10] and shoulder injury [11]. It is possible that this is in part driven by the observation that, in the US, Black males are more likely than White males to participate in football, basketball and soccer during preadolescence and adolescence, which are major sport contributors to the presenting injuries [12].

Another factor to consider is the socioeconomic status of these patients based on race, with known disparities likely contributing to both less resources and protective equipment available and therefore increasing injury likelihood. Given the high proportion of patients that identified as Black, it is possible that the sampling was skewed because there was a non-significant proportion of patients identifying as ‘Other’. This is indeed a limitation of large retrospective national databases, and it limits the inferences one can make on a national level in terms of race as a risk factor for HLC in this setting. Given that our regression analysis did not identify race as a predictor of HLC, it still remains a possibility that particular races may be more vulnerable for HLC; however, the current heterogeneity in the national definition and reporting of this issue is not statistically powerful enough to detect this.

The large majority of neck injury diagnoses were either strain (including sprain) or pain. These diagnoses, typically in the absence of more structural injury, can be treated conservatively, and as such most likely drive the high proportion of patients that were fortunately able to be discharged from the ED. The pediatric age group is particularly vulnerable to these diagnoses, in part due to a relatively less-mature musculoskeletal system within the neck [13]. Examples of this in the growing cervical spine of the neck include increased muscle and tendon tightness, less stable cartilage and a lagging bone mineralization behind linear bone development [14].

However, there was a small proportion of patients requiring HLC—these corresponded to the more serious and less common diagnoses of neck contusions (including abrasion), neck fractures and then neck lacerations. Sporting activities remain one of the primary causes of pediatric cervical fractures and these other serious neck injuries [15], and as such, this small proportion is not negligible. The extremes to which these injuries can affect patients neurologically include spinal cord injury, concussion, paralysis and death, and systemically include acute respiratory distress syndrome, anemia and shock [16].

Our analyses showed that that older age, male gender, involvement of body part(s) and non-sprain neck injuries all independently predicted the need for HLC in this setting. No specific neck injury type (e.g., strain vs. fracture) was found to be predictive of HLC in this study. Similar trends have been found previously in children with other sport injury types. Older age has been associated with poorer outcomes for shoulder dislocations [17], male gender is a greater risk factor for anterior cruciate ligament (ACL) tears in sports [18] and concomitant lower-extremity injury, head injury and thoracic injury all predisposed sport-related pediatric spinal injury patients to longer lengths of stay and need for intensive care [5]. Although the trends we report herein come from national data, future studies of more granular institutional data will be able to further triage how sensitive and specific these findings are to neck injuries.

The utility of our findings also includes the insight that sports contributing to these presentations can be gender-specific, which has been highlighted before [19]. For example, males playing football and females participating in cheerleading were the most commonly associated sports per gender, and did not feature in the other genders’ top ten contributors. This demonstrates that primary prevention should be targeted by proportions. That is to say, even though cheerleading is the sixth most common sport contributing to these neck injury presentations overall, it should be considered an important sport to target in females as it is the most common contributing sport in females. By highlighting these gender disparities, it is our hope that better allocations of effort can be made to globally reduce and prevent serious injuries for all pediatric participants as much as possible. Similar trends have been shown in the setting of sports-related pediatric concussions, highlighting the need to be even more aware of this [7].

There are limitations to this study. The first is that it is a retrospective review of prospectively gathered data from EDs only. Cases of suspected neck injuries diagnosed and treated on-field, or in a non-ED setting, would not have been captured in this database. Therefore, it is possible that our findings under-estimate the true incidence of these injuries. Another limitation is that these national estimates derive from a sample of EDs across the country. These de-identified datapoints then prevent quality assessment for patients with more than one presentation through the sample period to different hospitals or in different years. Although the focus of this study was to evaluate the trends of presentations independent of repeat presentations, it would be valuable in a future study to assess with more institutional data predictors of repeat presentations of these injuries.

Next, the definitions utilized by the NEISS are limited in their generalization of cervical injuries, given that this database is used for all emergency presentations. For example, the use of pain as a diagnosis can be argued to be limited given that pain is also a symptom. Pain after neck injury is assumed to be neck pain; however, this is highly subjective. There is no evidence of radiologic workup to diagnose sprains, such as X-ray for fractures and MRI for local edema, which may qualify as other diagnoses in other centers. It is also common in pediatric patients to have pseudolisthesis in the upper cervical spine as well as immature ossification centers, which if interpreted incorrectly can be reported as subluxation, lesional or as a fracture. It is difficult using these retrospective data to understand if any of these specific pediatric concerns have inflated or skewed the reported national trends. As such, the trends in reported neck injuries may not reliably reflect diagnoses specific to the pediatric cervical spine, and more robust criteria specific to the neck are needed in the future.

Finally, one must consider the heterogenous nature of ED care and these niche injuries. The decision to admit and pursue HLC is ultimately one made by the practicing ED physician, and it is highly likely that physician judgement can vary over time, as well as the judgement between physicians. This decision to admit may be further complicated by other factors such as available support at home that day or night and the desires and wishes of the patient and family. As such, our findings should be considered more as national trends rather than specific scenarios to account for this intrinsic individualized element in neck injuries that do present to the ED.

## 5. Conclusions

In this study, we demonstrate that a significant number of ED presentations for pediatric neck injuries during sporting activities occur annually in the United States. A non-negligible minority of patients will require HLC, and this requirement can be associated with older age, male gender, concomitant injuries and non-sprain injuries. Predictors were identified, as well as a number of gender-specific sports that associate with these presentations. These data demonstrate that there is a need for a greater institutional-level understanding of how these injuries present in order to better inform ED protocols in the future.

## Figures and Tables

**Table 1:** Clinical characteristics of entire cohort.

Parameter	Count (95% CI)	% Total (95% CI)
Age (mean)	12.7 (12.6–13.0)	
Age group		
Child	97,541 (68,700–126,000)	27.0% (24.8–29.4)
Preadolescent	130,970 (101,300–160,600)	36.3% (34.5–38.1)
Adolescent	132,373 (103,883–160,864)	36.7% (34.6–38.8)
Gender		
Male	216,490 (167,000–265,900)	60.0% (58.8–61.1)
Female	144,394 (108,801–180,000)	40.0% (38.9–41.2)
Race		
Black	182,548 (141,000–224,000)	50.6% (38.7–62.3)
White	116,304 (42,900–189,600)	32.2% (19.3–48.6)
Other	62,031 (40,500–83,500)	17.2% (11.9–24.1)
Location *		
Sporting complex	163,446 (122,200–204,600)	62.9% (57.9–67.5)
School	62,556 (43,153–81,960)	24.1% (20.1–28.5)
Home	33,917 (26,600–41,200)	13.1% (10.8–15.7)
Diagnosis		
Strain	216,249 (157,100–275,300)	59.9% (55.1–64.5)
Pain	105,100 (79,000–131,100)	29.1% (24.8–33.8)
Contusion (including abrasion)	23,252 (17,900–28,600)	6.4% (5.7–7.3)
Fracture	6192 (4400–7900)	1.7% (1.3–2.2)
Laceration	2932 (1800–4000)	0.8% (0.5–1.2)
Injury type		
Neck only	273,998 (208,200–339,800)	75.9% (73.0–78.6)
Neck and other body part(s)	86,900 (64,800–109,000)	24.1% (21.4–27.0)
Disposition		
Treated and released	344,517 (262,000–426,900)	95.5% (94.5–96.2)
Higher-level care (HLC) **		
Admission	6711 (4400–9022)	1.9% (1.3–2.5)
Transfer	5726 (3200–8200)	1.6% (1.0–2.4)
Observation	3318 (2300–4300)	0.9% (0.6–1.3)
Death	610 (190–1000)	0.2% (0.1–0.3)

* When reported; ** % overall for HLC was 4.5% (95% CI 3.8–5.4).

**Table 2:** Ranking of 10 most common sports associated with presentations overall, and by gender.

Rank	Overall	% Total (95% CI)	Males	% Total (95% CI)	Females	% Total (95% CI)
1	Football	18.6% (8.4–16.9)	Football	30.0% (27.4–32.8)	Cheerleading	12.3% (10.5–14.4)
2	Trampoline *	9.9% (8.9–11.1)	Trampoline	9.8% (8.4–11.5)	Trampoline	10.1% (9.1–11.2)
3	Basketball *	6.1% (5.4–6.9)	Basketball	6.9% (6.0–7.9)	Gymnastics	7.2% (6.2–8.3)
4	Soccer *	4.7% (3.9–5.6)	Wrestling	6.6% (5.5–8.0)	Soccer	5.1% (4.1–6.4)
5	Wrestling	4.4% (3.6–5.3)	Soccer	4.4% (3.5–5.4)	Basketball	4.9% (4.0–5.8)
6	Cheerleading	5.0% (4.2–5.9)	Cycling	4.2% (3.5–5.1)	Horse riding	4.1% (3.2–5.1)
7	Cycling *	3.7% (3.2–4.4)	Baseball	3.2% (2.7–3.7)	Softball	3.8% (2.7–5.1)
8	Gymnastics	3.3% (2.9–3.9)	Weightlifting	2.0% (1.6–2.5)	Cycling	3.0% (2.4–3.8)
9	Baseball	2.7% (1.5–4.5)	Ice hockey	1.3% (0.6–2.7)	Dancing	2.9% (2.1–3.9)
10	Athletics	2.1% (1.7–2.4)	Lacrosse	1.1% (0.7–1.6)	Volleyball	2.0% (1.5–2.6)

* Sports that ranked in the top 10 for both males and females.

## Data Availability

The original contributions presented in this study are included in the article. Further inquiries can be directed to the corresponding author.
